# Influence of Running Phases on the Postural Balance of Modern Pentathlon Athletes in a Laser Run Event

**DOI:** 10.3390/ijerph16224440

**Published:** 2019-11-12

**Authors:** Dorota Sadowska, Małgorzata Lichota, Tomasz Sacewicz, Justyna Krzepota

**Affiliations:** 1 Department of Physiology, Institute of Sport–National Research Institute in Warsaw, 01-982 Warsaw, Poland; 2 Department of Posture Correction and Compensation, Faculty of Physical Education and Sport in Biała Podlaska, Józef Piłsudski University of Physical Education in Warsaw, 21-500 Biała Podlaska, Poland; 3 Department of Biomechanics and Computer Science, Faculty of Physical Education and Sport in Biała Podlaska, Józef Piłsudski University of Physical Education in Warsaw, 21-500 Biała Podlaska, Poland; 4 Faculty of Physical Culture and Health, University of Szczecin, 71-065 Szczecin, Poland

**Keywords:** combined event, shooting position, modern pentathlon, body sway, fatigue

## Abstract

*Background*: The Laser Run combined event is part of the modern pentathlon, consisting of successive shooting and running phases. The main factor hindering accurate and fast shooting is the increasing fatigue caused by running effort. The aim of this study was to assess the impact of the running phases on the postural balance in the shooting position of pentathletes in a Laser Run event. *Methods*: 25 modern pentathletes (18.6 ± 1.7 years), members of the Polish Association of Modern Pentathlon, completed a Laser Run event. During each shooting series, a Zebris dynamometric platform recorded the displacement of the centre of pressure (COP). *Results*: Significant changes in the average velocity of the COP (F = 3.43; *p* = 0.0223) and the width of the ellipse of the COP shifts area WoE (F = 3.30; *p* = 0.0259) between the first and the second shooting series were observed. The average velocity of the COP in series I was 72.6 m/s and increased to 84.3 m/s in series II. In turn, the average width of the ellipse of the COP in series I reached 29.1 mm and in series II, 34.1 mm. *Conclusions*: The fatigue caused by the running phases in the Laser Run affects the stability of the shooting position of pentathletes. Disturbances that occur after the first running phase are maintained at the same level during the subsequent shooting series. The fatigue level does not affect the magnitude of the disturbances of the postural balance in the shooting position.

## 1. Introduction

A Laser Run combined event is part of the modern pentathlon consisting of shooting and running phases, each of which the athlete performs four times. The results obtained in the Laser Run are an important element that often determines the outcome of the modern pentathlon event [1,2]. The factor that hinders accurate and fast shooting is the increasing fatigue caused by covering the subsequent running distances.

Running is considered to be one of the physical activities that affects postural balance the most [3]. This is because running engages most of the body’s muscle groups, including the muscles responsible for maintaining postural stability and, more than other activities, affects the sensory receptors (especially the muscle spindles, joint receptors, and cutaneous mechanoreceptors on the feet), which leads to a deterioration of their sensitivity [4,5].

Previous studies demonstrated that a single bout of physical effort and the resulting fatigue contribute to a decrease in athletes’ postural balance and negatively affect their performance. These unfavourable effects of physical effort have been, inter alia, documented in football players [6,7], judo athletes [8], taekwondo athletes [9], and in the biathlon [10,11,12,13], which, similarly to the Laser Run, is composed of alternating effort and shooting phases. A significant decrease in the shooting performance and postural balance among biathletes was observed after progressive intensity cycle ergometer exercise [13], intense skiing exercise [14], and roller skiing on a treadmill [12]. Moreover, in our previous studies, we observed a deterioration in the postural balance and rifle stability in the standing shooting position in biathletes both after moderate intensity physical efforts [11] and after maximum physical effort [10]. At the same time, we found a strong correlation between postural sways and rifle sways during targeting [10,11].

It should be noted that the shooting position of biathletes and that of pentathletes differ significantly. The specific body position during standing shooting in the biathlon allows for putting the centre of gravity of the rifle closer to the medium line of the body and locating the rifle above the centre of the support area, which provides stability to the position. In turn, pentathletes hold the weapon only with one completely outstretched hand. The weight of the arm and the pistol will substantially disturb the postural balance. The athlete has to bend the upper part of the body backwards to provide a counterweight to the weight of the extended arm with the pistol and regain the optimal position of the body’s centre of gravity. The only reports in the literature on the postural balance and shooting performance of pentathletes have been presented by Dadswell et al. [1,15]. In their first article, the authors compared the shooting precision of modern pentathletes and pistol shooters. The results showed that while the pistol shooters achieved significantly greater scores than modern pentathletes under precision conditions, there were no differences between the groups under fatigue conditions. In the following studies conducted among pentathletes, the authors observed that in the combined event played according to the modern pentathlon rules pre-2013, the displacements of the centre of pressure (COP) after a short sprint of 20 m in the first shooting series are larger in comparison with those observed during the static stance, but do not change after covering the subsequent running distances.

Due to the dynamic development, increasing popularity, and significant changes that have been made in recent years in the Laser Run rules, and because of the lack of scientific reports on the factors that determine the results of the Laser Run, the aim of this study was to assess the impact of the running phases on the postural balance in the shooting position of pentathletes in the Laser Run event played in accordance with the current rules.

## 2. Materials and Methods

### 2.1. Participants

This study involved 25 pentathletes (12 women and 13 men; mean age: 18.6 ± 1.7 years) who were members of the Polish Association of Modern Pentathlon. All the athletes competed at the national or both the national and international levels. The body height and mass of the pentathletes were 177.1 ± 8.3 cm and 66.5 ± 8.2 kg, respectively. 

Written informed consent was sought from all participants or legal guardians, in the case of underage subjects. The protocol of the study conformed to the recommendations of the Declaration of Helsinki and was approved by the local Bioethics Committee (decision 113 no. KEBN-18-41-MŁ).

### 2.2. Procedure

Prior to the study, each participant was provided with detailed information about the test procedures and the research methodology. Each participant was examined individually. 

Testing took place at a shooting range, conforming to ISSF shooting regulations. The sequence of tasks followed the order detailed by the highest level of competition of the Laser Run organized by The Union Internationale de Pentathlon Moderne (2018) [16] (Table 1). Participants began the Laser Run with a short 20 m sprint that preceded the first shooting series and were instructed to complete each phase at a pace similar to that which they would use in a competition. 

Postural balance was examined using the Zebris FDM-2 Force Distribution Measuring System from the Body Posture Laboratory at the Regional Centre for Research and Development of the University College in Biała Podlaska. The Zebris platform features individually calibrated motion detectors, which make it possible to analyse the density distribution of static and dynamic forces acting on the ground while standing and also record the centre of pressure (COP) signal. As the subject stood on the platform (dimensions: 212 × 60.5 × 2.1 cm; number of miniature force sensors: 15,360), the force exerted by their feet was recorded by the sensors at a sampling rate of 120 Hz. The postural balance was evaluated four times, during each shooting series. The coordinates of the instantaneous COP were calculated with WinPDMS processing software v1.2.1 (Zebris GmbH, Isny, Germany) (Table 2).

In the Laser Run, the number of shots an athlete can take to achieve the 5 hits within the 70 s time limit is unlimited. Therefore, participants took a varied number of shots within each series. The lowest number of shots a participant could shoot was 5 if all the shots were accurate. Consequently, the analysis of the COP displacement was based on the first 5 shots of each series to ensure homogeneity and that appropriate data were available for comparisons.

### 2.3. Statistical Analysis

Statistical analyses were conducted using Statistica 13.0 software (Dell Inc. Dell Statistica) [17]. The normality of distribution of the variables analysed was verified using the Shapiro–Wilk test. The data were analysed using ANOVA with four repeated measures (series I, series II, series III, series IV) and Bonferroni post hoc tests were applied for the analysis of any significant results. The statistical significance was set at *p* < 0.05. In the case of multiple comparisons, the Bonferroni correction was applied to reduce the chances of obtaining false-positive results.

## 3. Results

**Figure 1:**
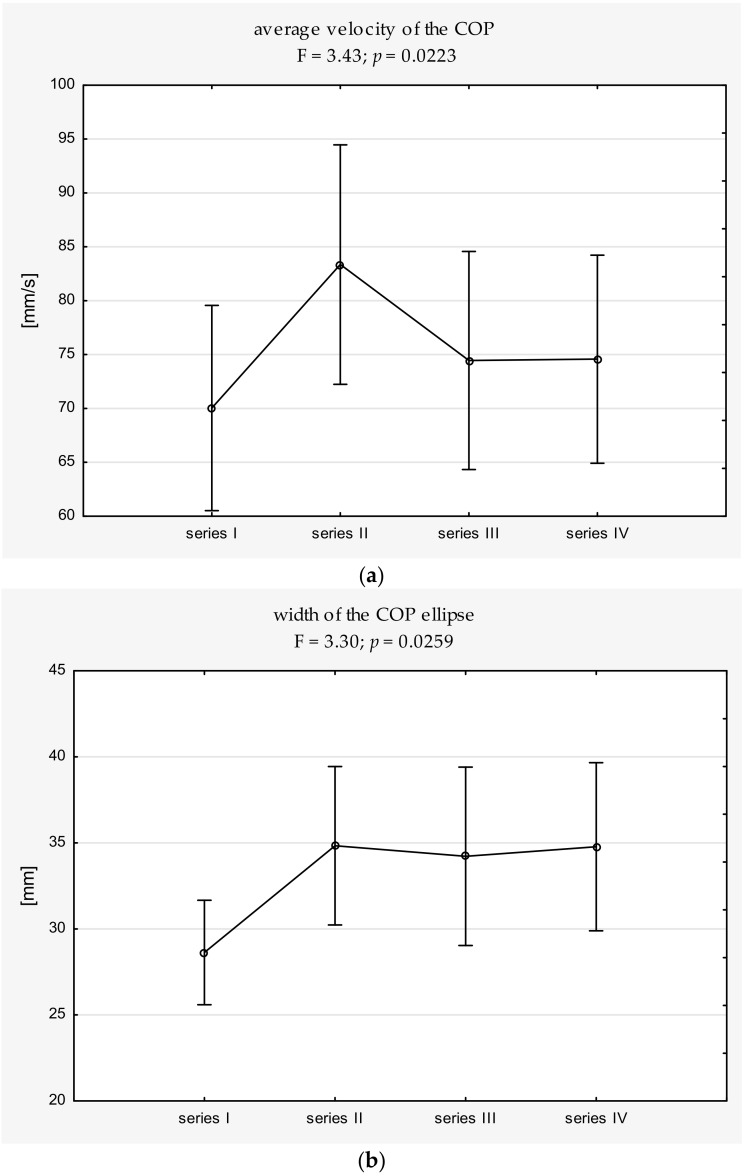
Mean values and 95% confidence intervals for the COP measures: (**a**) average velocity (V) and (**b**) width of the ellipse (WoE) in each shooting series, along with the results of ANOVA with repeated measures. Significant differences (adjusted *p* value to 0.0083) are marked with *. * adjusted *p* ≤ 0.0083.

ANOVA revealed significant changes in V (F = 3.43; *p* = 0.0223) and the width of the ellipse (WoE) (F = 3.30; *p* = 0.0259) between series (Table 3). The V and WoE increased significantly between shooting series I and series II (Figure 1). The average velocity of the COP in series I was 72.6 m/s and increased to 84.3 m/s in series II. In turn, the average width of the ellipse of the COP in series I reached 29.1 mm, and, in series II, 34.1 mm. There were no further changes in the above parameters in the subsequent shooting series. Neither the height of the ellipse (HoE) nor the area of the centres of pressure (AoE) changed significantly between the series.

## 4. Discussion

The results of our study indicate that running fatigue during the Laser Run has a significant impact on the athlete’s postural balance in the shooting position. The results do not confirm Dadswell’s reports [1], in which there were no increased postural sways during shooting after consecutive distances of 2 km. It should be noted that Dadswell [1] used a different test protocol and different measuring instruments (two AMTI force platforms). In our research, the athletes shot four times (series I–IV) and covered the distances of 800 m in accordance with the current rules of the highest level of competition of the Laser Run organized by the Union Internationale de Pentathlon Moderne (2018). In addition, we used a large enough platform, which allowed the athletes to adopt a comfortable shooting position, as they do in a competition. It was important to us that the athletes could take a comfortable and natural foot position during each shooting series. According to the Hawkins and Sefton [18] study, the appropriate width of foot spacing can improve postural stability and shooting performance. 

Our results indicate a noticeable decrease in the postural balance after covering the first 800 m of the running distance (during the II shooting series) in relation to the level presented by the athletes after a short 20 m sprint (during I series of shooting). Interestingly, however, the values of the analysed postural sway parameters did not change significantly with the increasing fatigue; that is, after subsequent running sections. This implies that the magnitudes of the postural balance disturbances were essentially the same regardless of fatigue level. Similar results were recorded in our earlier studies of biathletes. Physical effort caused a significant deterioration in the stability of the standing shooting position, but an increase in the intensity of the effort did not affect the further increase in the postural balance disturbances.

In our research, we observed an increase in the COP velocity caused by physical effort. Similar results—the increased velocity of the COP—were recorded by Derave et al. [19] after walking and running on a treadmill; Marcolin et al. [3] after moderate treadmill running, and Guidetti et al. [20] after prolonged treadmill running exercise at individual ventilatory and anaerobic thresholds. According to Corbail et al. [21], the faster instantaneous oscillations, which are manifested by an increase in the COP velocity, could be associated with the discrete control of the postural oscillations required to compensate the motor and sensory deficiencies induced by peripheral muscular fatigue.

What is interesting is the visible decline and then the maintenance of a constant level of the COP velocity during the III and IV series of shooting. This may be related to the adaptation of the postural balance system to the fatigue. However, the lack of statistically significant changes does not allow us to draw such conclusions and this issue requires further research.

Another parameter of which the values increased significantly after covering the first running distance was WoE. WoE reflects the length of the ellipse of the COP oscillation towards the athlete’s mediolateral (ML) direction. However, we did not observe any significant changes in the anteroposterior (AP) direction. The most important role in the shooting process is played by the arm holding the pistol, which is outstretched in the athlete’s frontal plane. When assuming the shooting position, the weight of the arm and the pistol will dramatically disturb the postural balance, and high levels of muscular forces and the engagement of other parts of the body are necessary to regain the most optimal position of the centre of mass. General muscular exercise, e.g., running, generates physiological disturbances and alterations of sensory receptors and motor output for postural function [5], affects movement organization, and significantly increases reaction time [22]. In fatigue conditions, the central nervous system is not able to compensate for the disturbances in the postural function and the postural balance is thus disturbed [5]. We assume that the specific position of the athlete’s body, which requires from the neuromuscular system the allocation of more effort to control mediolateral stability than anteroposterior stability, is responsible for the disturbances noted in the ML direction.

To sum up, the running effort in the Laser Run affects the stability of the athletes’ shooting position. Disturbances in the postural balance that occur after the first running phase are maintained at the same level during the subsequent series of shooting, which indicates that the level of fatigue does not affect the magnitude of the postural balance disturbances in the shooting position. Results of earlier studies conducted among shooters [23] and biathletes [10,11] indicate a strong correlation between the postural balance and rifle stability. It can therefore be assumed that postural balance disturbances caused by physical effort may have a negative impact on the shooting accuracy of pentathletes. Since shooting accuracy was not assessed in this study, this issue requires further research. 

## Figures and Tables

**Table 1:** The sequence of tasks in the Laser Run during the testing in accordance with The Union Internationale de Pentathlon Moderne (2018).

Running Sequences	Total Distance	Shooting Sequences	Distance to the Targets
4 × 800 m	3200 m	4 × 5 hits	10 m

**Table 2:** A specification of the analysed posturographic measures.

Indicator	Description of the Measures
*COP shifts*	
V [mm/s]	Average velocity of the COP
*Surface area of the COP*	
AoE [mm^2^]	Area of the centres of pressure (calculated from the COP shifts in such a way that 95% of the data are within the ellipsoid and 5% are outside)
WoE [mm]	Width of the ellipse (the length of the ellipse in mediolateral direction)
HoE [mm]	Height of the ellipse (the length of the ellipse in anteroposterior direction)

**Table 3:** Means and standard deviations for posturographic measures recorded in each shooting series along with the results of ANOVA with repeated measures.

Parameter	Series I	Series II	Series III	Series IV	F	P
	Mean (SD)	Mean (SD)	Mean (SD)	Mean (SD)		
V [mm/s]	72.6 (23.1)	84.3 (26.3)	75.7 (25.0)	75.3 (22.7)	3.43	0.0223
AoE [mm^2^]	1369.9 (674.6)	1663.6 (839.3)	1567.6 (605.6)	1512.0 (846.6)	1.39	0.2552
WoE [mm]	29.1 (6.9)	34.1 (10.1)	34.4 (11.2)	34.1 (10.8)	3.30	0.0259
HoE [mm]	57.8 (20.9)	59.9 (20.3)	65.8 (23.9)	53.3 (16.9)	1.37	0.2589
